# Enhanced field emission performance of gold nanoparticle decorated Bi_2_S_3_ nanoflowers†

**DOI:** 10.1039/d4na00539b

**Published:** 2024-11-13

**Authors:** Gorkshnath H. Gote, Madhura P. Deshpande, Somnath R. Bhopale, Mahendra A. More, Raphael Longuinhos Monteiro Lobato, Jenaina Ribeiro-Soares, Dattatray J. Late

**Affiliations:** a Sir Parashurambhau College (Autonomous), Department of Physics Pune-411030 India; b Department of Chemistry, Research Institute of Chem-Bio Diagnostic Technology, Chung-Ang University 84 Heukseok-ro, Dongjak District Seoul 06974 Republic of Korea; c Centre for Advanced Studies in Materials Science and Condensed Matter Physics, Department of Physics, Savitribai Phule Pune University Pune-411007 India mam@physics.unipune.ac.in; d Departamento de Física, Universidade Federal de Lavras Campus Universitário, PO Box 3037 Lavras Minas Gerais 37200-000 Brazil jenaina.soares@ufla.br datta099@gmail.com; e School of Physics, IISER Thiruvananthapuram Thiruvananthapuram Kerala 695551 India

## Abstract

Au nanoparticles (NPs) are decorated on hydrothermally synthesized Bi_2_S_3_ nanorods (NRs) to enhance the field electron emission (FEE) performance as compared to bare Bi_2_S_3_ nanorods, resulting in reduction in turn-on field from 3.7 to 2.7 V μm^−1^ (at the current density of 1.0 μA cm^−2^) with significant increment in maximum emission current density from 138 to 604.8 μA cm^−2^ (at a field of 7.8 V μm^−1^) respectively. FESEM/TEM reveals that Bi_2_S_3_ nanoflowers are assembled from Bi_2_S_3_ NRs of a typical diameter of 120 ± 10 nm, and Au NPs of diameter about 5–10 nm are uniformly decorated onto the surface of NRs to form an Au/Bi_2_S_3_ composite. XRD analysis suggests that the as-synthesized product consists of orthorhombic Bi_2_S_3_ NRs decorated with face-centered cubic Au NPs. The XPS spectrum shows the elemental mapping of the as-synthesized Au/Bi_2_S_3_. Improvement in field emission properties is mainly attributed to a reduction in work function and increasing emitting sites due to Au NP decoration.

## Abstract 2

Gold nanoparticles are decorated on Bi_2_S_3_ nanorods to improve field electron emission performance, lowering the turn-on field and increasing the emission current density.

## Graphical abstract

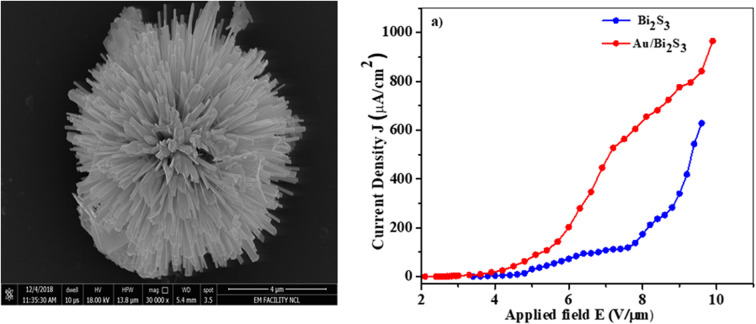

## 1 Introduction

Field emission is a quantum mechanical tunneling effect in which electrons are emitted from a metal/semiconductor surface into a vacuum under a sufficiently intense electric field. FEE cathodes possess diverse advantages over thermionic emitters with respect to durability, current density, and low energy consumption, which place FEE devices in the competition for next-generation electronics. FEE cathodes are widely used in certain electron-beam devices, such as flat panel displays,^1^ scanning electron microscopes,^2^ and X-ray sources.^3^ The FEE properties of emitters are related to their composition, tip sharpness, conductivity, field enhancement factor, and work function. To increase the field enhancement factor β it is required to reduce tip sizes and modify the work function, by doping, decorating or preparing composites of nanostructured materials. In this sense, much effort has been made to study nanostructure cathodes for FEE applications such as LaB_6_ nanowires,^4^ carbon nanotubes (CNTs),^1,5^ ZnO,^6^ TiS nanosheets,^7^ as well as composites such as CNTs-LaB_6_,^8^ rGO-ZnS,^9^ rGO-ZnO,^10^ Au-ZnO,^11^*etc.* have been reported as possible field emitter materials.

**Fig. 1:**
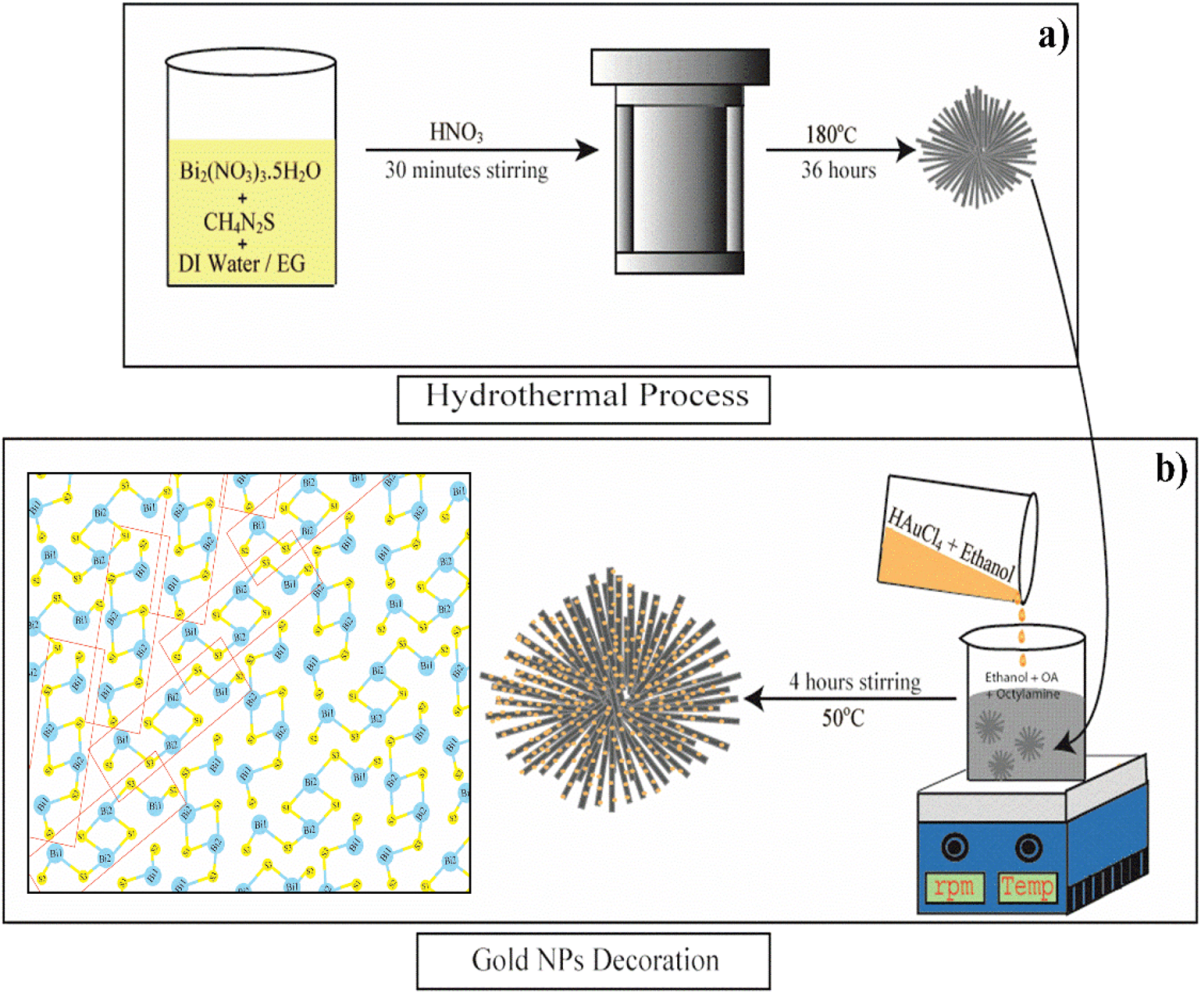
Schematic depiction of the mechanism of (a) the hydrothermal synthesis of Bi_2_S_3_ microflowers, (b) Au decoration process on as-synthesized Bi_2_S_3_ NRs and schematic structure of Bi_2_S_3_.

Since the last few years, metal sulfides have been extensively studied due to their tunable optical and electronic properties, so they are extensively used in energy conversion and storage device applications such as solar cells,^12^ supercapacitors,^13^ Na-ion batteries,^14^*etc.* Among various metal sulfides, Bi_2_S_3_ is the most extensively researched direct bandgap semiconductor. As shown in Fig. 1b, the Bi_2_S_3_ crystal structure is formed by the polymerization of the tightly bonded [Bi_4_S_6_] unit, where each unit is connected by weak Bi–S and S–S interaction.^15^ The benefits of low cost, abundance, non-toxicity, and excellent optoelectronic and electrical properties make Bi_2_S_3_ an excellent material for practical applications, such as in photodetectors,^16^ solar cells,^17^ photocatalysis,^18,19^ supercapacitors,^20^*etc.* Improvement in FEE performance was mainly attempted by increasing the field enhancement factor (depending on the shape/morphology of the emitters) and reducing the work function of nanostructured emitters. In order to improve FEE performance, it is preferred to study composite/heterostructure properties of nanostructured metal sulfides. Recently, number of investigations were made to improve the FEE performance by blending rGO,^21,22^ metal oxides,^23,24^ sulfides,^25,26^ and materials with various metal sulfides of 1D/2D nanostructures. In addition, many efforts have been made to study the morphology-dependent FEE performance of Bi_2_S_3_ nanostructures.^27–30^ However, FEE improvement by work function modulation is less explored for the Bi_2_S_3_ nanostructure. Our group has investigated the FEE performance of CdS-Bi_2_S_3_ (ref. 25) and rGO-Bi_2_S_3_ (ref. 31) composites.

Noble metal NPs (Au, Ag, and Pt) on semiconductors have been extensively studied and exploited in several applications, such as photocatalysis,^32,33^ solar cells,^34,35^ light-emitting diodes,^36^ photodetectors,^37,38^ FETs,^39^*etc.* These reports suggest that the decoration of noble metals on nanomaterials enhances the performance of bare nanomaterials due to the Surface Plasmon Resonance (SPR) phenomenon. In the literature survey, it was found that the properties of FEE emitters were greatly influenced by the surface decoration of various morphology nanostructures (ZnO-nanopillars,^11^ CdO nanosheets,^40^ Si nanowires,^41^ SiC nanowires,^42^ and graphene sheets^43^) with Au NPs, owing to increase in emitting sites and decrease of the work function. Au metal is well known for its low resistivity, high oxidation resistance, and high structural, electrical and chemical stability. The influence of the decoration of Au NPs on the properties of nanostructures of Bi_2_S_3_ FEE emitters has not been reported to date.

It has been observed from a literature survey that most nanostructured FEE emitters are decorated with Au NPs by a sputtering process.^11,40–42,44^ In this report, we follow a cost-effective and simple chemical method to decorate Au NPs on hydrothermally synthesized Bi_2_S_3_ NRs to explore the FEE performance. It is observed that the turn-on field of Au/Bi_2_S_3_ NRs drastically decreased from 3.7 to 2.7 V μm^−1^ at the current density of 1 μA cm^−2^ and the maximum current density increased from 138 to 604.8 μA cm^−2^ at an applied field of 7.8 V μm^−1^ as compared to that of pristine Bi_2_S_3_ NRs. Enhancement in FEE characteristics indicates that metal NP decoration could be an effective route to significantly enhance the FEE performances of the Bi_2_S_3_ cathodes.

## 2 Experimental

### 2.1 Synthesis of Bi_2_S_3_ nanoflowers

Bismuth sulfide (Bi_2_S_3_) micro-flowers were synthesised as per the literature report with slight modifications.^45^ In a typical synthesis, 2.4 g of bismuth nitrate [Bi(NO_3_)_3_·5H_2_O] (bismuth precursor) and 0.38 g of thiourea [CS(NH_2_)_2_] (sulfur precursor) were dissolved well into a mixture of 45 ml distilled water and 20 ml ethylene glycol (EG). The resultant solution was subjected to 30 minutes of stirring and 10 minutes of ultra-sonication at room temperature. This yellow homogeneous mixture was then transferred into a Teflon-lined stainless steel autoclave of 80 ml capacity and a few drops of concentrated HNO_3_ were added to it. The sealed autoclave was then kept at 180 °C for 36 hours in a muffle furnace. After the reaction, the resulting black precipitate was thoroughly washed (three times) with distilled water and absolute ethanol and collected by centrifugation (4000 rpm for 10 min). The final product was dried at 60 °C for 24 h in a vacuum. The schematic representation of the Bi_2_S_3_ microflower synthesis process is shown in Fig. 1a. The resultant black powder was used for further characterization and Au decoration.

### 2.2 Synthesis of Au/Bi_2_S_3_ nanostructures

Bi_2_S_3_ NRs were decorated with Au NPs by following a modified literature report.^46^ Briefly, 82 mg of as-synthesized Bi_2_S_3_ microflowers was dispersed in a 30 ml solvent mixture of 24 ml ethanol, 1.4 ml oleic acid (OA) and 4 ml octylamine. At the same time, 34 mg tetrachloroauric(iii) acid (HAuCl_4_·3H_2_O) was dispersed into 10 ml of ethanol. Then, the gold precursor solution was added dropwise to the above mixture and stirred for 4 hours at about 50 °C. The grey precipitation was washed several times with ethanol, separated by centrifugation, and then dried overnight in a vacuum. The schematic representation of Au decoration of Bi_2_S_3_ NRs is shown in Fig. 1b. The collected sample (Au/Bi_2_S_3_) was used for further characterization and FEE comparison studies with pristine Bi_2_S_3_.

### 2.3 Field electron emission measurements

The FEE characteristics of pristine and Au/Bi_2_S_3_ samples were investigated at room temperature using an in-house developed setup. The distance between emitter sites (cathode) and phosphor screen (anode) is kept at 2 mm, with base pressure being maintained at ∼10^−8^ mbar during measurements. A high voltage power source (Spellman, U.S.) was used to supply the voltage between two electrodes. The voltage was increased by a step of 20 V and the corresponding increasing current was measured using an electrometer (Keithley 6514) with picoampere sensitivity.

## 3 Characterization of materials

To examine the crystalline phases of both structures, the X-ray diffraction pattern was obtained by using a Rigaku MicroMax-007 HF with a rotating anode copper X-ray source of wavelength *λ* Cu Kα = 1.54 Å which was operated at 40 kV and 30 mA. The morphological and structural characterizations were done using a field emission scanning electron microscope (FESEM, FEI Nova Nano SEM450) and transmission electron microscope. Further structural details and lattice fringe width calculations of both samples were accomplished by high-resolution transmission electron microscopy (HRTEM TEM, JEOL JEM-F200 operated at 200 kV accelerating voltage). X-ray photoelectron spectroscopy was achieved with the use of a near-ambient-pressure X-ray photoelectron spectrometer (XPS, Thermo K-Alpha^+^ Spectrometer using Al-K_α_ X-rays, 1486.6 eV) under ultra-high vacuum conditions to study elemental compositions of as-synthesized structures.

## 4 Results and discussion

### 4.1 XRD analysis

**Fig. 2:**
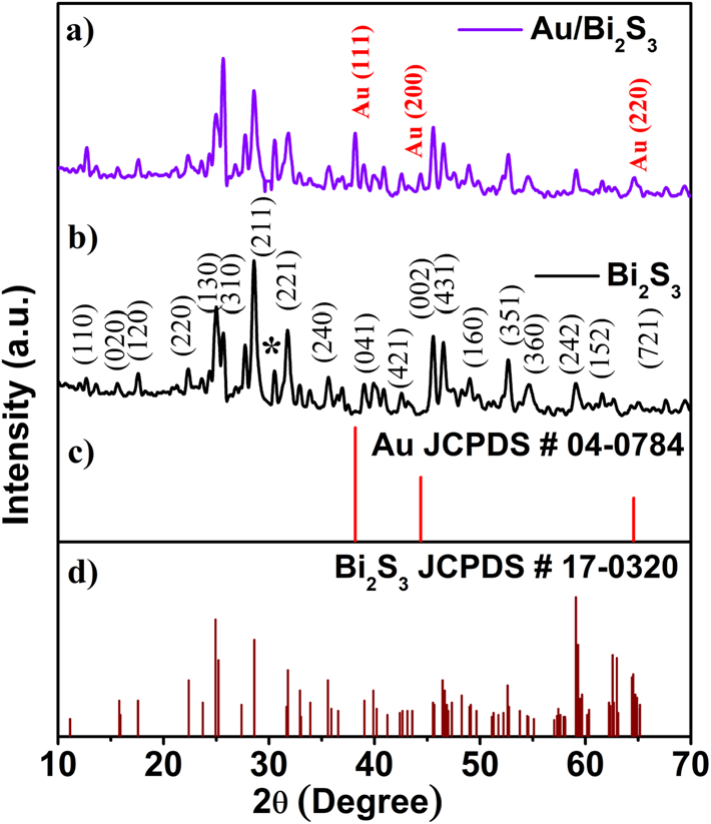
XRD of the (a) Au/Bi_2_S_3_ sample and (b) Bi_2_S_3_; (c) and (d) are JCPDS cards of Au and Bi_2_S_3_ crystals, respectively.

The phase and crystallinity of samples were studied by the X-ray diffraction technique. Fig. 2a and b show the typical XRD pattern of the as-prepared Au NP decorated Bi_2_S_3_ and pristine Bi_2_S_3_ sample respectively. The XRD pattern illustrates that the synthesized sample has an orthorhombic crystal structure with lattice parameters *a* = 3.981 Å, *b* = 11.14 Å, *c* = 11.30 Å (Fig. 2d, JCPDS # 17-0320) which also existed in the Au/Bi_2_S_3_ sample. The average crystallite size of Bi_2_S_3_ NRs was found to be ∼110 ± 10 nm, as calculated using the Debye–Scherer equation. Fig. 2a displays a peak at about 2*θ* = 38.10°, corresponding to the (111) plane of face-centered cubic (fcc) gold, (JCPDS # 04-0784), which gives the evidence for uniform gold decoration. The peak at about 2*θ* = 30.50° can be attributed to the characteristic peak of BiS_2_ (JCPDS #17-0267) indicating the negligible amount of BiS_2_ in the as-synthesized samples.

### 4.2 FESEM and TEM analysis

**Fig. 3:**
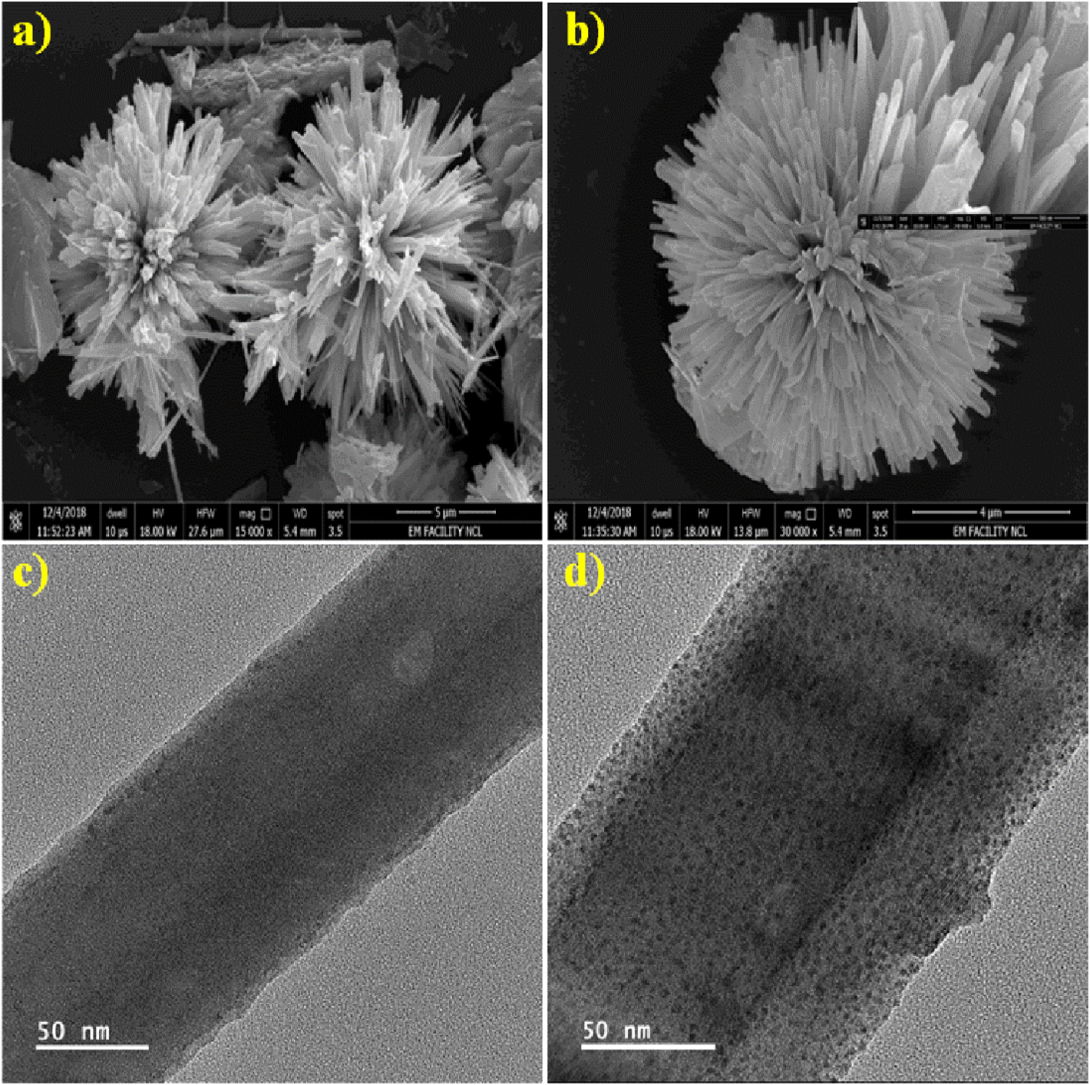
FESEM images: (a) low magnification Bi_2_S_3_ microflowers, (b) high magnification Bi_2_S_3_ with the inset displaying nanorods; TEM images (c and d) of bare Bi_2_S_3_ and Au decorated Bi_2_S_3_ NRs, respectively.

**Fig. 4:**
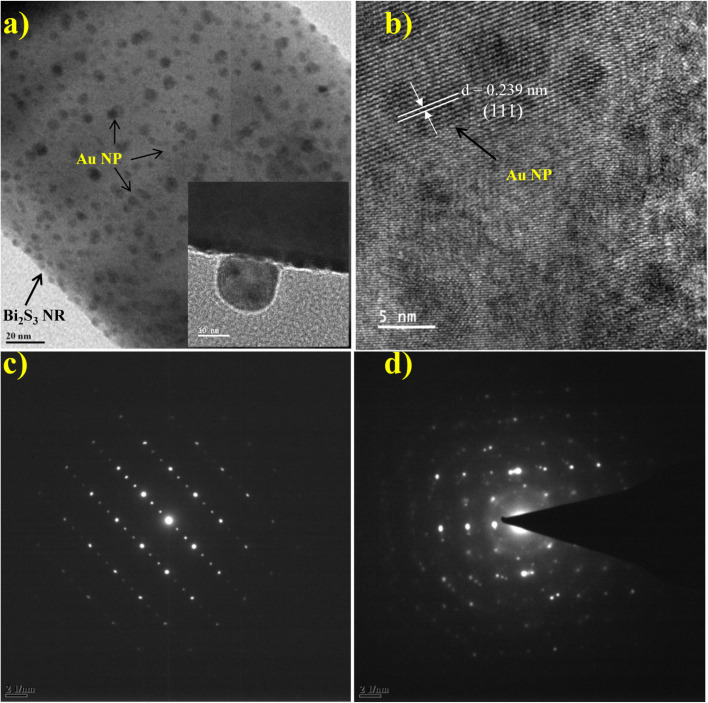
TEM image of (a) Au/Bi_2_S_3_ NRs with the inset displaying the NPs; (b) HRTEM image of Au/Bi_2_S_3_; (c and d) SAED pattern of bare Bi_2_S_3_ NRs and Au/Bi_2_S_3_ NRs, respectively.

FESEM and TEM were used to examine surface morphology, size, and Au particle distribution on the as-prepared sample. Fig. 3a depicts an overview of the sample, where the Bi_2_S_3_ micro-flowers are observed with approximately ∼4 to 5 μm diameter. Fig. 3b shows typical images assembled from the number of NRs. The inset in Fig. 3b is the high -magnification FESEM image of Bi_2_S_3_ micro-flowers. It is seen from the TEM image (Fig. 3c) that bare Bi_2_S_3_ NRs have a smooth surface with length in few microns and about ∼120 nm in diameter, which is also endorsed by the average crystallite size calculated from the XRD pattern using the Debye–Scherer equation. The typical enlarged TEM image (Fig. 3d) of the Au decorated Bi_2_S_3_ nanorods reveals that the Au NPs were uniformly decorated over the entire surface of the Bi_2_S_3_ NRs without extended agglomerations. The high-resolution TEM (HRTEM) images displayed in Fig. 4a illustrate that the decorated Au NPs have an average diameter of 4–10 nm. The fine HRTEM fringes (Fig. 4b) reveal that the distance between two consecutive planes is 0.239 nm which is identical to the interplanar spacing of face-centered cubic (fcc) Au(111) planes.^47^Fig. 4c and d display the Selected Area Electron Diffraction (SAED) pattern of pristine Bi_2_S_3_ and Au/Bi_2_S_3_ NRs.

### 4.3 XPS analysis

**Fig. 5:**
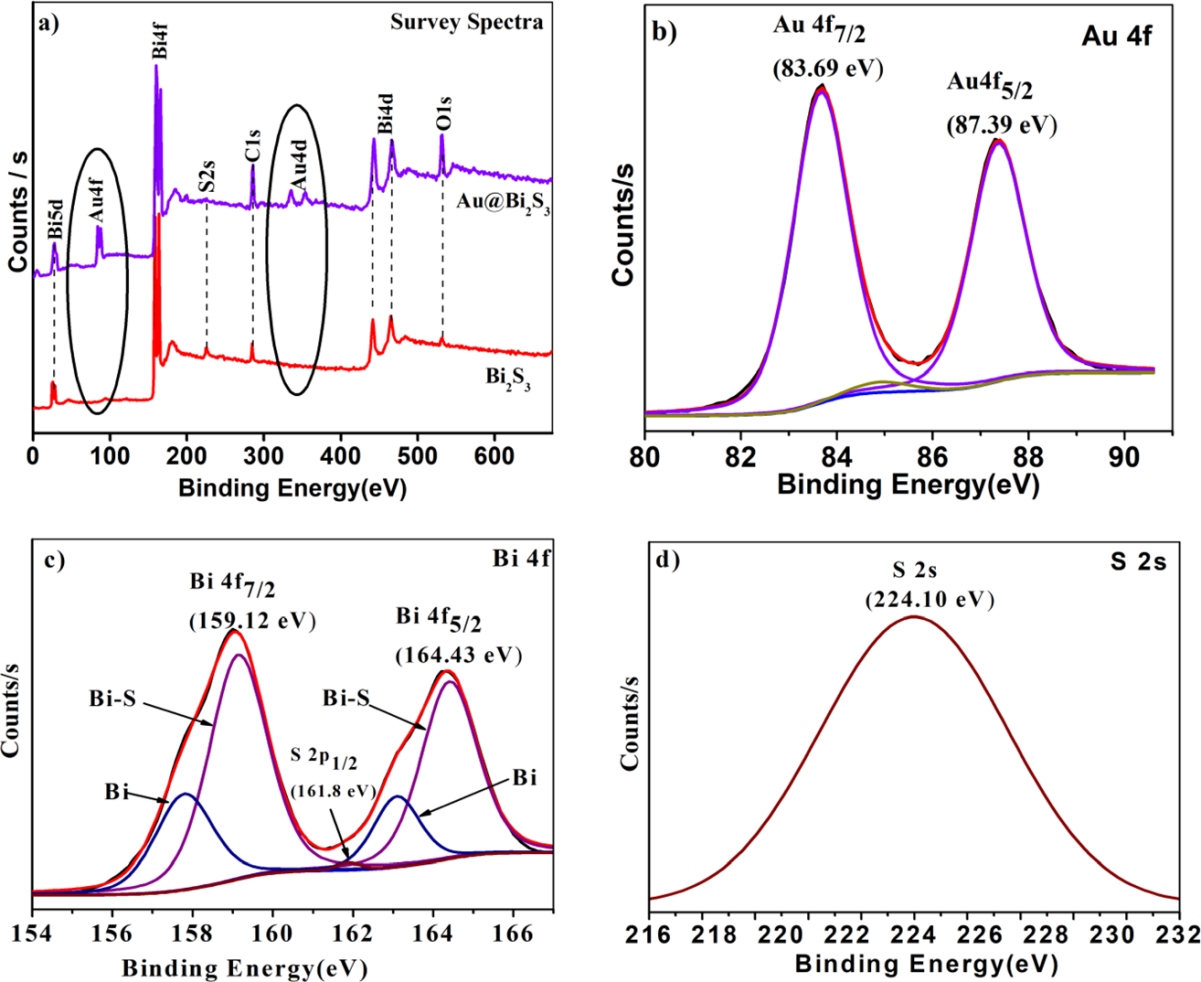
XPS images of (a) Bi_2_S_3_ and Au/Bi_2_S_3_ survey spectra; high resolution spectra of (b) Au^0^ state, (c) Bi^3+^ state, (d) S^2−^ state of the Au/Bi_2_S_3_ sample.

Surface chemical compositions and oxidation states of the as-synthesized product were investigated by XPS analysis. Fig. 5a shows the survey spectra of the sample with and without Au decoration, and gives strong evidence for the existence of Bi and S elements along with the successful decoration of Au on Bi_2_S_3_. The O and C peaks also arise due to the adsorbed oxygen species on the sample surface, which is commonly observed for samples exposed to the atmosphere and adsorbed carbon species during XPS measurement respectively. Adventitious C1s (284.6 eV) spectra were taken as the reference for calibration. Au 4f_7/2_ and 4f_5/2_ doublets with binding energies of 83.69 and 87.39 eV are observed in Fig. 5b, which asserts that decorated Au (Au^0^ state) is in the metallic form (ref. 48 and 49). As shown in Fig. 5c, the high-resolution spectrum of Bi_4f_ (Bi^3+^ state) shows a doublet at 159.14 (4f_7/2_) and 164.39 eV (4f_5/2_) attributed to spin–orbital coupling separated by 5.31 eV.^49^ The binding energy for S2s (S^2−^ state) (Fig. 5d) in the Au/Bi_2_S_3_ sample is at 224.1 eV which is lower than the binding energy of S2s (S^2−^ state) (225.7 eV) in pristine Bi_2_S_3_ as per the literature.^49^ The Bi and S binding energies are in good agreement with reported values. It is observed that the Au decoration does not severely change the crystallinity of Bi_2_S_3_ NRs. All XPS plots validate the successful formation of Au/Bi_2_S_3_.

### 4.4 Reaction mechanism

In our earlier report, we discussed the synthesis mechanism of Bi_2_S_3_ microflowers prepared by a simple one-pot hydrothermal method for FEE studies.^31^ For the decoration of Au NPs on Bi_2_S_3_ NRs, HAuCl_4_·3H_2_O was decomposed at 50 °C in an octylamine–oleic acid mixture under ambient conditions. In this reaction, the octylamine–oleic acid mixture acts as both a capping and a reducing agent, which prevents gold NP aggregation and oxidation, as well as makes the particle surface hydrophobic.^50^ The polar ethanol solvent was primarily used to dissolve HAuCl_4_·3H_2_O. The presence of the hydrocarbon surfactant between gold particles possibly prevents the growth of particles beyond 5–7 nm.^51^ To ensure the even mixing of the reactants, the solution was continuously stirred beyond the decoration time and also this ensured the uniform deposition of Au NPs onto the Bi_2_S_3_ surface as seen in TEM images (Fig. 3d). The pristine Bi_2_S_3_ and Au/Bi_2_S_3_ nanocomposites have been fully characterized and their FEE properties studied.

### 4.5 Field electron emission (FEE) performance

The dependence of FEE current density over an applied field (*J*–*E*) is described by the modified Fowler–Nordheim (F–N) equation:^52^1
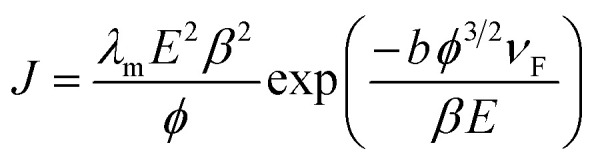
where *J* is the emission current density, *E* is the applied average electric field, *λ*_m_ is the macroscopic pre-exponential factor (=1.54 × 10^−6^ A eV V^−2^), *b* is a constant (=6.83 eV^−3/2^ V nm^−1^), *ϕ* is the work function of the emitter, *β* is the field enhancement factor, and *ν*_F_ (correction factor) is a particular value of the principal Schottky–Nordheim barrier function *ν*.

Eqn (1) asserts that FEE property enhancement can be achieved by either/both tuning the morphology or/and lowering the work function of the emitter. We have tried to improve the FEE performance of Bi_2_S_3_ NRs by Au decoration, resulting in reduced work function of Bi_2_S_3_.

**Fig. 6:**
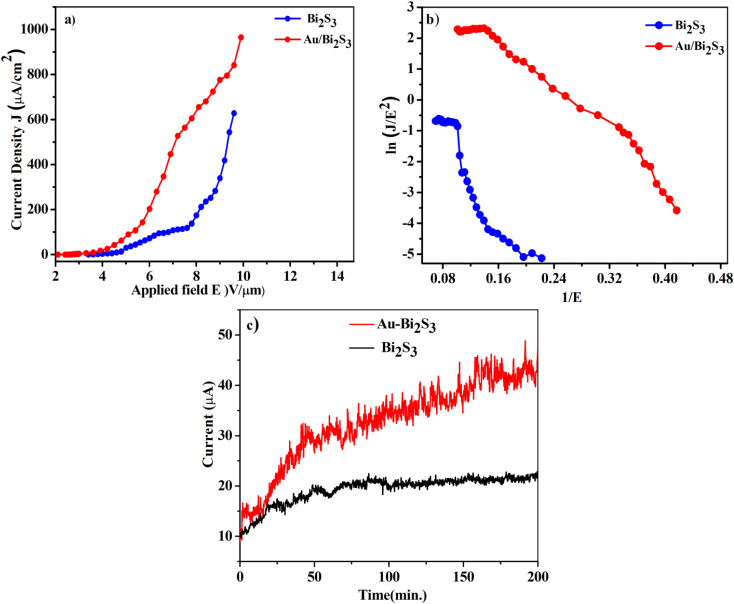
Plot of field emission current density *versus* applied electric field (*J*–*E*) (a), Fowler–Northeim (F–N) plot (b) of Bi_2_S_3_ and Au/Bi_2_S_3_, respectively. (c) Field emission current stability plots at 10 μA.

The turn-on and threshold fields, measured from the *J*–*E* plot (Fig. 6a), are arbitrarily defined at emission current densities of 1 and 100 μA cm^−2^ respectively. The Au/Bi_2_S_3_ emitters show the turn-on and threshold fields of 2.7 and 5.2 V μm^−1^ respectively, whereas pristine Bi_2_S_3_ emitters show the turn-on and threshold fields of 3.7 and 6.8 V μm^−1^ respectively. It is obvious from the results that the FEE properties of pure Bi_2_S_3_ can be dramatically improved by Au decoration.

A wide range of research groups have reported a significant enhancement in FEE performance due to Au decoration on semiconductor materials.^11,41–44^ Zang *et al.*^53^ reported that the reduction of Au NP size below 10 nm decreases the work function up to 3.6 eV. In this work, decorated Au NPs have an average size of 4–10 nm, indicating that the work function of Au NPs would be around 3.6 eV, which is less than the work function of Bi_2_S_3_ NRs (4.93 eV).^25^ When Au (metal) and Bi_2_S_3_ (semiconductor) come into contact, they form a metal–semiconductor junction (Schottky barrier junction). The thermal equilibrium has been achieved through the transfer of electrons from Au (higher) to Bi_2_S_3_ (lower). The bending of the conduction band (CB) and valence band (VB) in Bi_2_S_3_ takes place due to the alignment of work functions. This energy band bending reduces the work function of the Bi_2_S_3_ emitter. Such work function reduction, due to NP decoration of the nanomaterial, shows an improvement of the FEE properties.^54^

The F–N plots (Fig. 6b) derived from the observed (*J*–*E*) curves show deviation from the linear nature, in contrast to the expectation of the F–N model. Such discrepancy has been observed in a diverse range of metal–semiconductor composites. This discrepancy can be supported by emission from the lower edge of the CB being dominant at a low field. On the other hand, at a high field, the emission current also contributes to the electrons in the upper edge of the CB.^62^ It is observed that the nonlinearity of the F–N plot diminishes in the Au/Bi_2_S_3_ emitter, indicating a more metallic behavior of the emitter. Furthermore, a careful observation reveals ‘flattening’ of the F–N plots in the high field region. For planar emitters, most of the researchers have noticed a ‘non-linear’ nature of the F–N plots, along with the tendency to show ‘flattening’ in the high field region, which had been attributed to various effects like the field screening effect, field penetration and band bending (for semiconducting emitters), *etc.* However, one of the root causes of such discrepancy is failure of the fundamental F–N model. It has been realized that the effect and/or contribution due to space charge limited currents has been ignored. Various researchers have focused their studies towards amending the fundamental F–N equation so as to justify the FEE behaviour of planar emitters. Herein, a planar emitter is referred to as an assembly of nanostructures deposited in a thin film form on suitable substrates. In tune with the advancement in planar emitter based electron sources and new devices, these alterations are important in providing better formulations for simulations.

In this context, Richard Forbes has put in significant and consistent efforts towards modification of the fundamental F–N model.^52,63,64^ In tune with these efforts, Zhang *et al.* have presented an overview of the fundamental physics of space–charge interactions in various media addressing the critical developments on various theoretical aspects of the space–charge limited current (SCLC) model, its physics at the nanoscale, and transitions between electron emission mechanisms and material properties.^65^ Very recently, in order to corroborate the advancements in utilization of electron sources, particularly simulations of the devices, Kevin Jensen has provided useful formulations considering the effect of “space–charge” (commonly described by the Child–Langmuir law) so as to guarantee correct numerical evaluation of the fundamental equations describing the various electron emission models.^66^

Furthermore, to showcase the technological importance of the observed FEE parameters, an attempt is made to compare the values of turn-on and threshold fields along with maximum emission current density extracted from similar emitters, Table1.

**Table 1:** FEE characteristic parameters of various semiconducting planar emitters

Sr. no.	Emitter details	Morphology	Turn-on field or voltage	Reference
1	MoS_2_	Nanoflowers	12 V μm^−1^	55
2	CNTs	Nanotubes	50 to 100 V	56 and 57
3	Au/ZnO	Nanowires	6.2 V μm^−1^	58
4	AuBN	Nanocomposites	3.9 V μm^−1^ at 10 nA cm^−2^	59
5	Au/graphene	Nanosheets	3.84 V μm^−1^	60
6	Au/TiO_2_	Nanotubes	2.8 V μm^−1^ at 10 μA cm^−2^	61
7	Au/Bi_2_S_3_	Micro-flowers	2.7 V μm^−1^ at 1 μA cm^−2^	Present work

Finally, to test the quality of the as-synthesized Au/Bi_2_S_3_ emitters in comparison with pristine Bi_2_S_3_, we have investigated the long-term current stability (*I*–*t*) plot. The stability of the emitters has been observed for more than 3 h corresponding to a current density of 10 μA cm^−2^. It is observed that fluctuations in current density for the Au/Bi_2_S_3_ emitter (the standard deviation of ∼3.86%) are somewhat more than for the bare Bi_2_S_3_ (the standard deviation of about ∼2.07%), possibly due to increase in emitting sites for the Au/Bi_2_S_3_ emitter. The fluctuation in electron emission current can be attributed to the adsorption/desorption and bombardments of ions/atoms on the emitter surface.^67,68^ The smoothness in the current stability (*I*–*t*) plot of the Au/Bi_2_S_3_ sample is also attributed to the protection of Bi_2_S_3_ NR emitters from ion/atom bombardments during the FEE mechanism due to the good chemical stability of Au NPs. The emitter also reveals good repeatability of results by testing the same samples several times.

## 5 Conclusions

In summary, we have reported the synthesis of Bi_2_S_3_ emitters with 120 ± 10 nm diameter, *via* a simple hydrothermal route followed by the uniform decoration of Au NPs with size ∼4–10 nm. FEE results show that the turn-on field of the Au/Bi_2_S_3_ emitter reduced from 3.7 to 2.7 V μm^−1^ at 1.0 μA cm^−2^ and the maximum current density increased from 138 to 604 μA cm^−2^ at field 7.8 V μm^−1^ as compared to that of pristine Bi_2_S_3_. The improved FEE performance of Au/Bi_2_S_3_ is attributed to the lowering of the work function of pristine Bi_2_S_3_ and increasing emission sites due to Au NP decoration. Therefore, Au decoration on Bi_2_S_3_ was achieved by a simple and cost-effective chemical method compared to the sputtering process. The Au decorated FEE emitters can be used as a potential candidate for flexible flat panel displays, efficient electron guns, and e-paper applications.

## Data availability

All data generated or analyzed during this study are included in this published article and its ESI.†

## Author contributions

GG and MD performed the synthesis experiments, characterization, and analysis. FEE analysis was done by SB and MM. GG wrote the first draft of the paper. GG, DL, and MM revised the writing of the manuscript and data calculations. RLML and J. Ribeiro-Soares in the revised the writing of the manuscript and data calculations. DL guided the research and conceived the final project.

## Conflicts of interest

The authors declare no conflicts of interest.

## Supplementary Material

NA-OLF-D4NA00539B-s001
