# Supplementary material for: Enhanced field emission performance of gold nanoparticle decorated Bi2S3 nanoflowers
Source: Nanoscale Adv. 2024 Nov 13;7(1):310–9. doi: 10.1039/d4na00539b (PMC11603640; doi:10.1039/d4na00539b)
Supplement: NA-007-D4NA00539B-s001 [file NA-007-D4NA00539B-s001.pdf]

This journal is © The Royal Society of Chemistry 2024

Supporting Information

Figure S1:

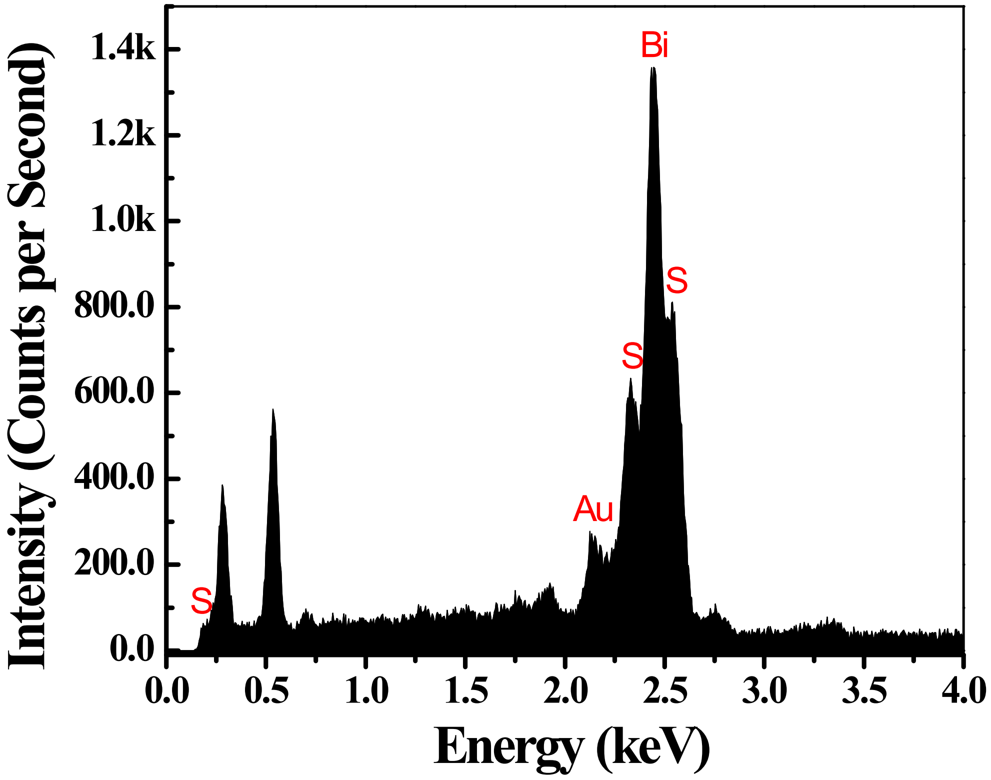

Sr. no.	Element	Weight%
1.	Au	8.20
2.	S	10.73
3.	Bi	81.07

Figure S1: EDAX analysis of Au nanoparticle decorated Bi_2S_3 nanoflower.

Figure S2:

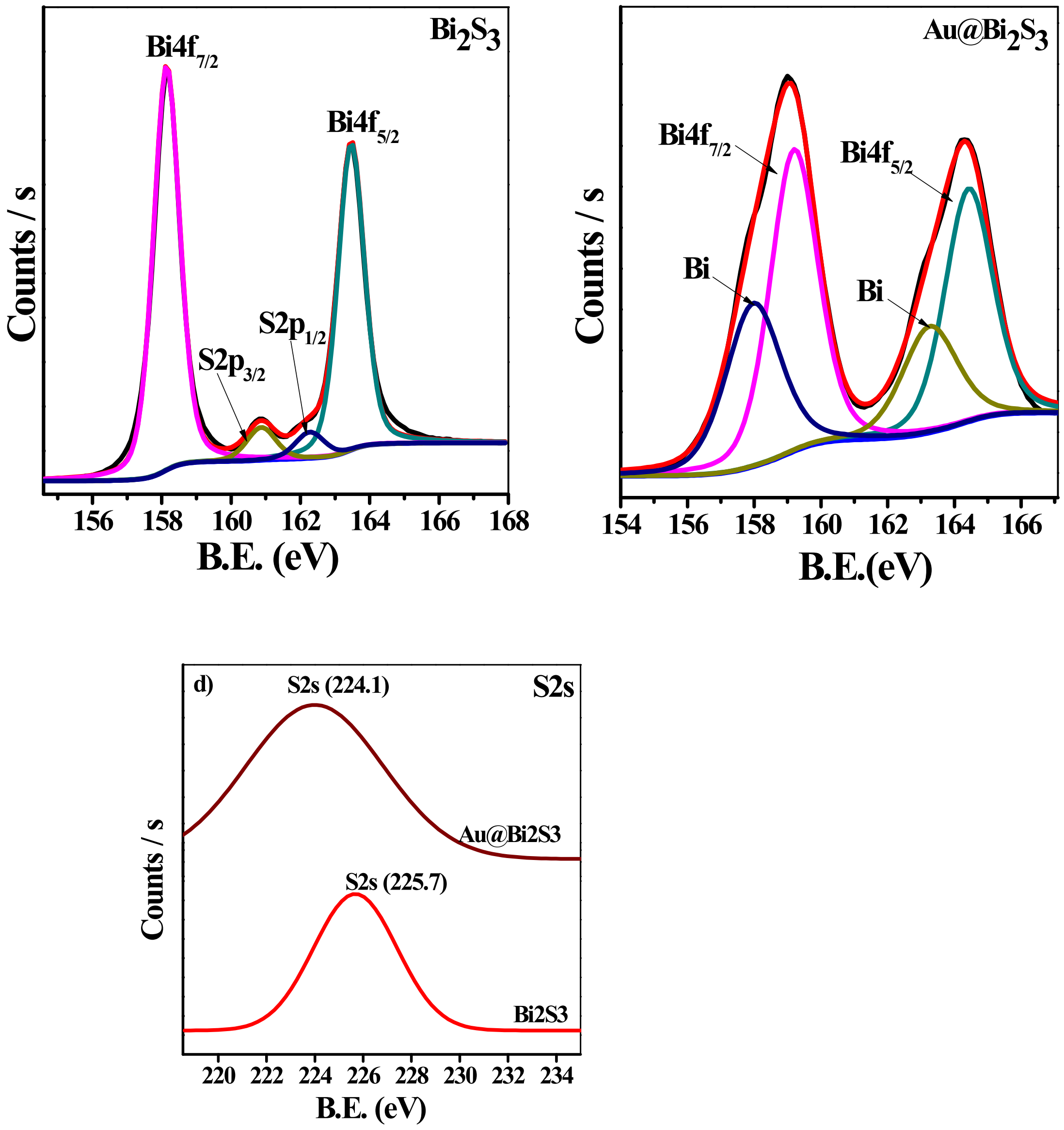

Figure S2: XPS deconvolution spectra for Bi2S3 nanoflower and Au decorated Bi2S3 nanoflower.

Figure S3:

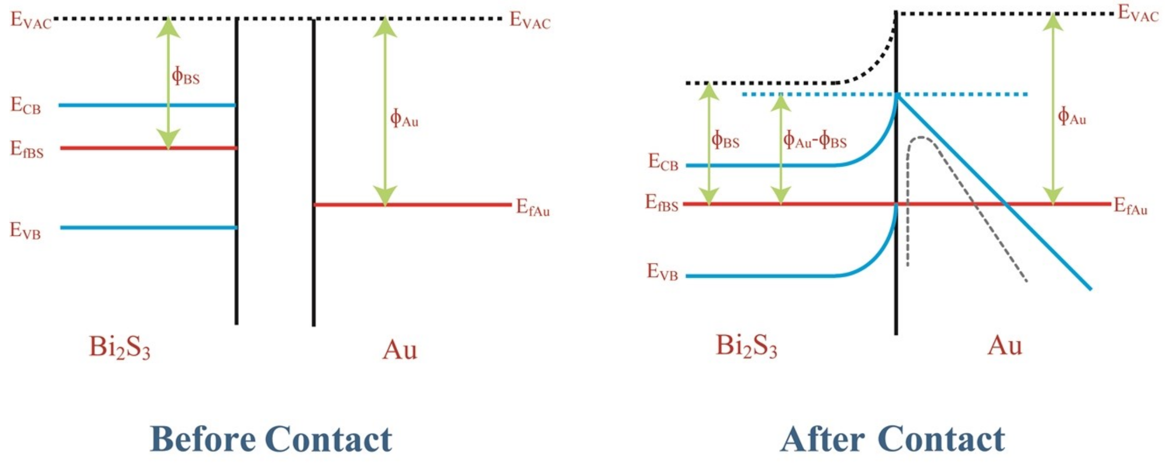

Figure S3: Field emission Energy band Diagram showing emission current tunneling from VB to CB at an intense electric field.
